# MNK1/2 inhibition limits oncogenicity and metastasis of *KIT*-mutant melanoma

**DOI:** 10.1172/JCI181338

**Published:** 2024-04-15

**Authors:** Yao Zhan, Jun Guo, William Yang, Christophe Goncalves, Tomasz Rzymski, Agnieszka Dreas, Eliza Żyłkiewicz, Maciej Mikulski, Krzysztof Brzózka, Aniela Golas, Yan Kong, Meng Ma, Fan Huang, Bonnie Huor, Qianyu Guo, Sabrina Daniela da Silva, Jose Torres, Yutian Cai, Ivan Topisirovic, Jie Su, Krikor Bijian, Moulay A. Alaoui-Jamali, Sidong Huang, Fabrice Journe, Ghanem E. Ghanem, Wilson H. Miller, Sonia V. del Rincón

Original citation: *J Clin Invest*. 2017;127(11):4179–4192. https://doi.org/10.1172/JCI91258

Citation for this corrigendum: *J Clin Invest*. 2024;134(8):e181338. https://doi.org/10.1172/JCI181338

The authors recently became aware that in the original Figure 2, A and C, the same eIF4E and GAPDH immunoblots were shown. The legend failed to indicate that these figure panels showed immunoblots from the same lysate, and in Figure 2C, the eIF4E blot for the MM111 D820Y panel was flipped horizontally.

The authors were able to provide immunoblots from the original data and have corrected Figures 2A and 2C to show loading controls for all gels presented in these figure panels and to show immunoblots that were run in parallel within each panel. The authors have also provided the unedited blot and gel images for all immunoblots and gels in the manuscript and supplement. The corrected figure panels and updated figure legend appear below.

The authors regret the errors.

## Supplementary Material

Unedited blot and gel images

## Figures and Tables

**Figure 2:**
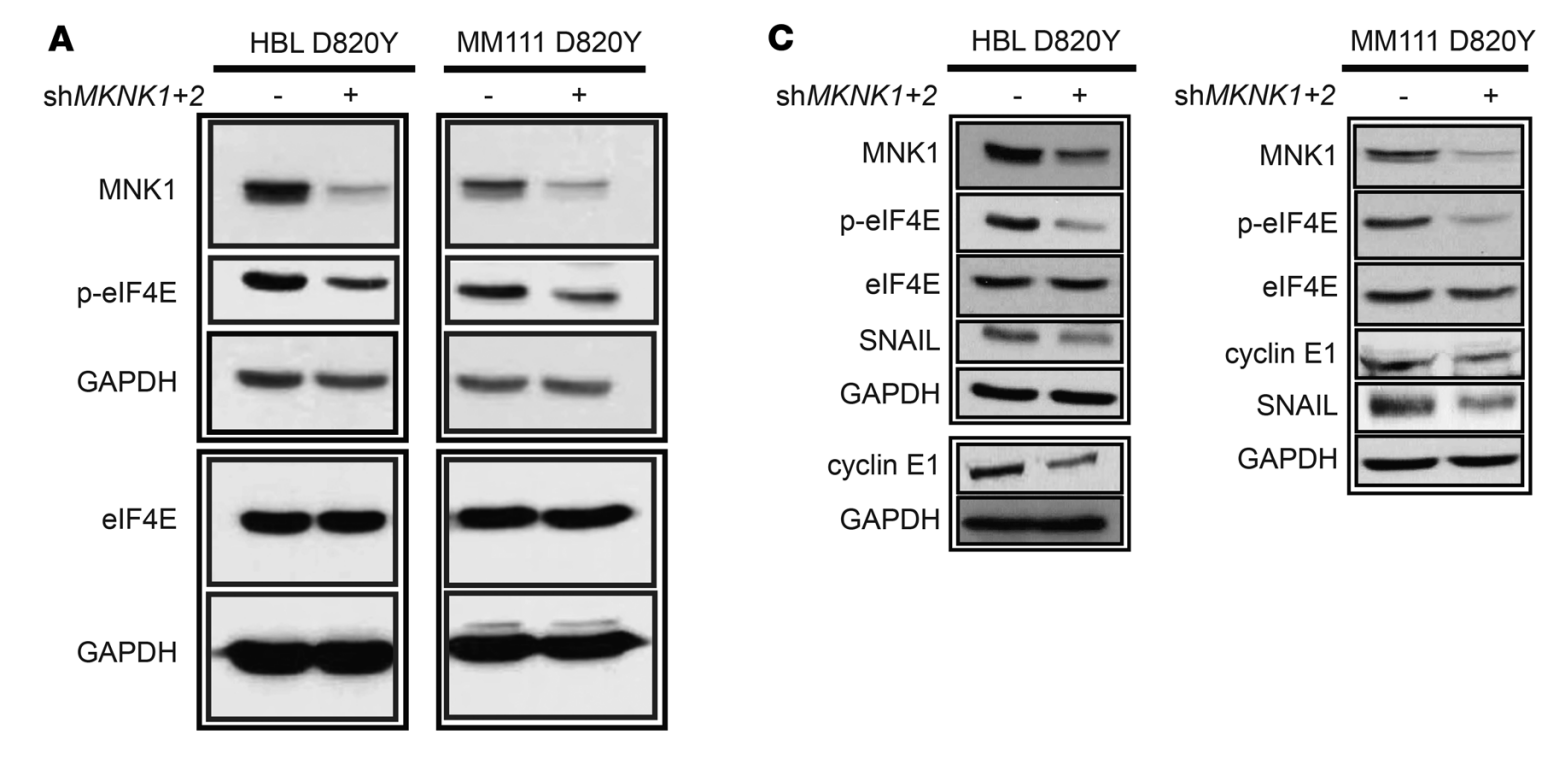
MNK1/2 knockdown in HBL cells suppresses cell migration and the expression of cyclin E1 and SNAIL. (**A**) Western blot analysis of MNK1, p-eIF4E, and eIF4E in HBL or MM111 cells expressing shCTL and sh*MKNK1*+2 (left). RT-qPCR was performed to examine the expression level of *MKNK2* mRNA in HBL and MM111 cells expressing shCTL and sh*MKNK1*+2 (right). (**B**) Cell migration was assessed by Transwell assay in shCTL versus sh*MKNK1*+2 HBL and MM111 cells after 48 hours. Representative images are shown. Scale bars: 200 μm; original magnification, ×10. (**A** and **B**) Data represent the mean ± SD, *n* = 3. **P < 0.01 by 2-tailed Student’s *t* test. (**C**) Western blot analysis of MNK1, p-eIF4E, eIF4E, cyclin E1, and SNAIL in HBL and MM111 shCTL and sh*MKNK1*+2 cell lines. (**A** and **C**) GAPDH is used as loading control. Panels **A** and **C** show Western blot data from lysate derived from the same experiment.

## Version 1 04/15/2024

Electronic publication

