# Supplementary material for: MNK1/2 inhibition limits oncogenicity and metastasis of KIT-mutant melanoma
Source: J Clin Invest. 2024 Apr 15;134(8):e181338. doi: 10.1172/JCI181338 (PMC11014651; doi:10.1172/JCI181338)

Figure 1A

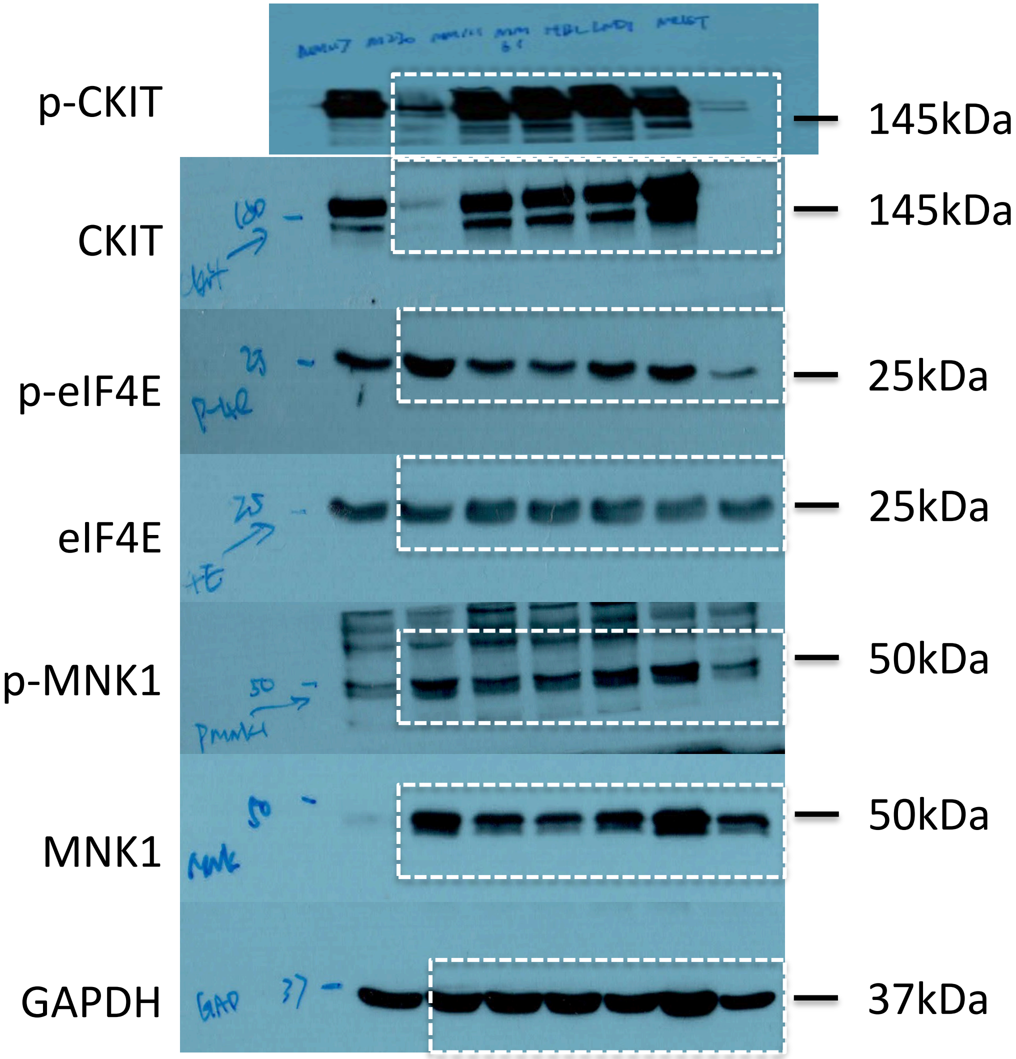

p-CKIT
CKIT
p-eIF4E
eIF4E
p-MNK1
MNK1
GAPDH
145kDa
145kDa
25kDa
25kDa
50kDa
50kDa
37kDa

Figure 1C

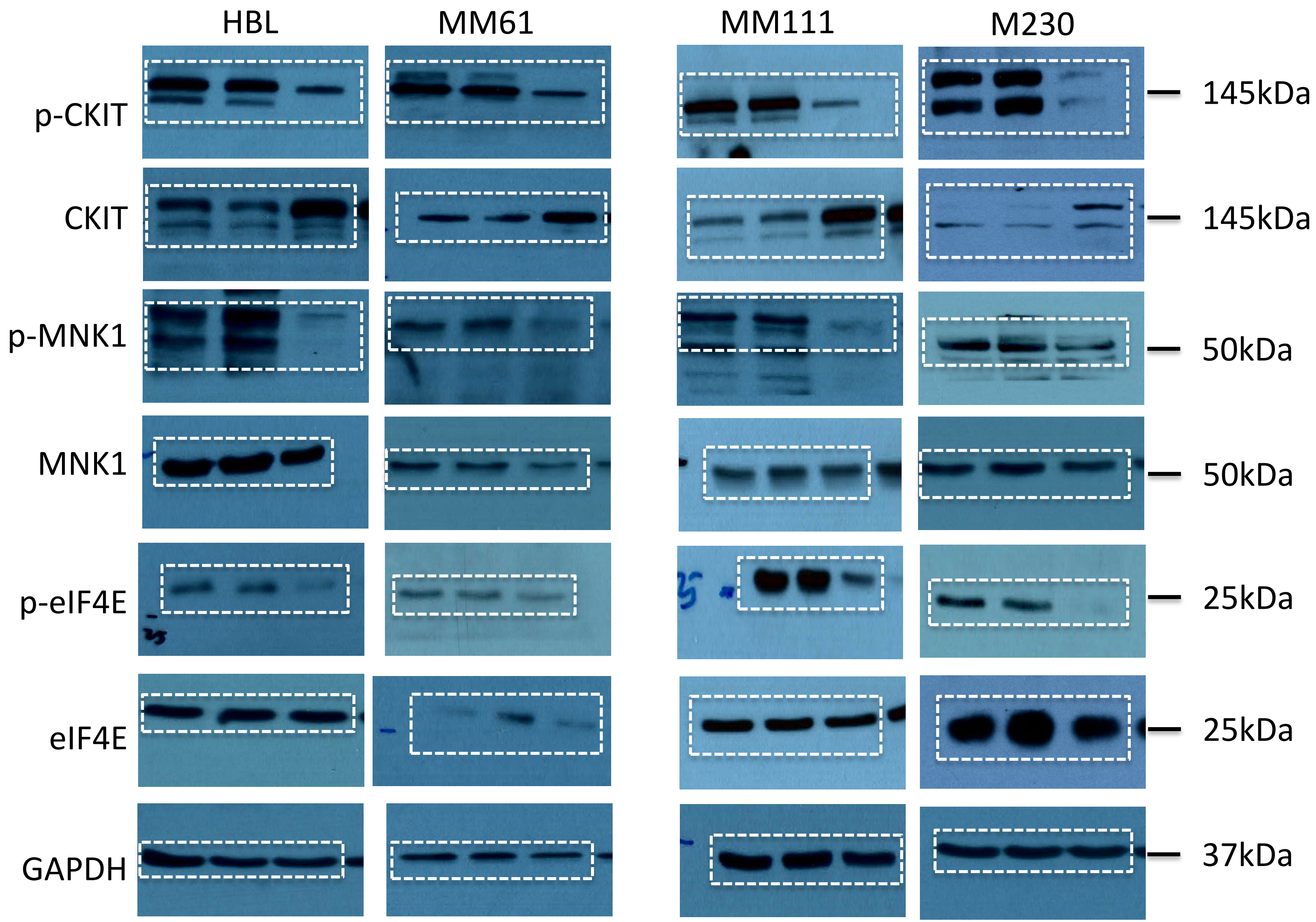

HBL
MM61
MM111
M230
p-CKIT
CKIT
p-MNK1
MNK1
p-eIF4E
eIF4E
GAPDH
145kDa
145kDa
50kDa
50kDa
25kDa
25kDa
37kDa

Figure 1E

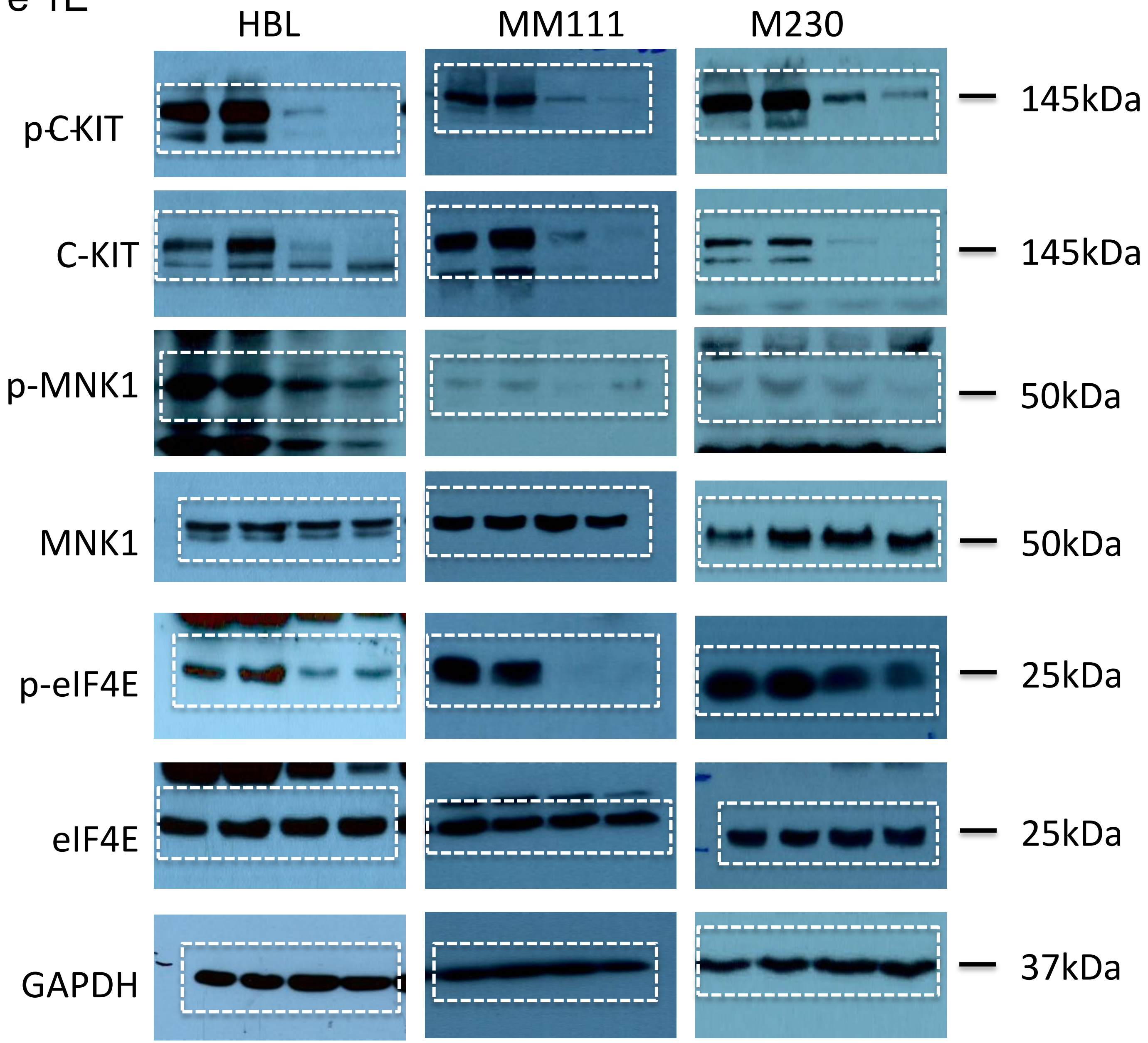
HBL
MM111
M230
p-C-KIT
C-KIT
p-MNK1
MNK1
p-eIF4E
eIF4E
GAPDH
145kDa
145kDa
50kDa
50kDa
25kDa
25kDa
37kDa

Figure 2A

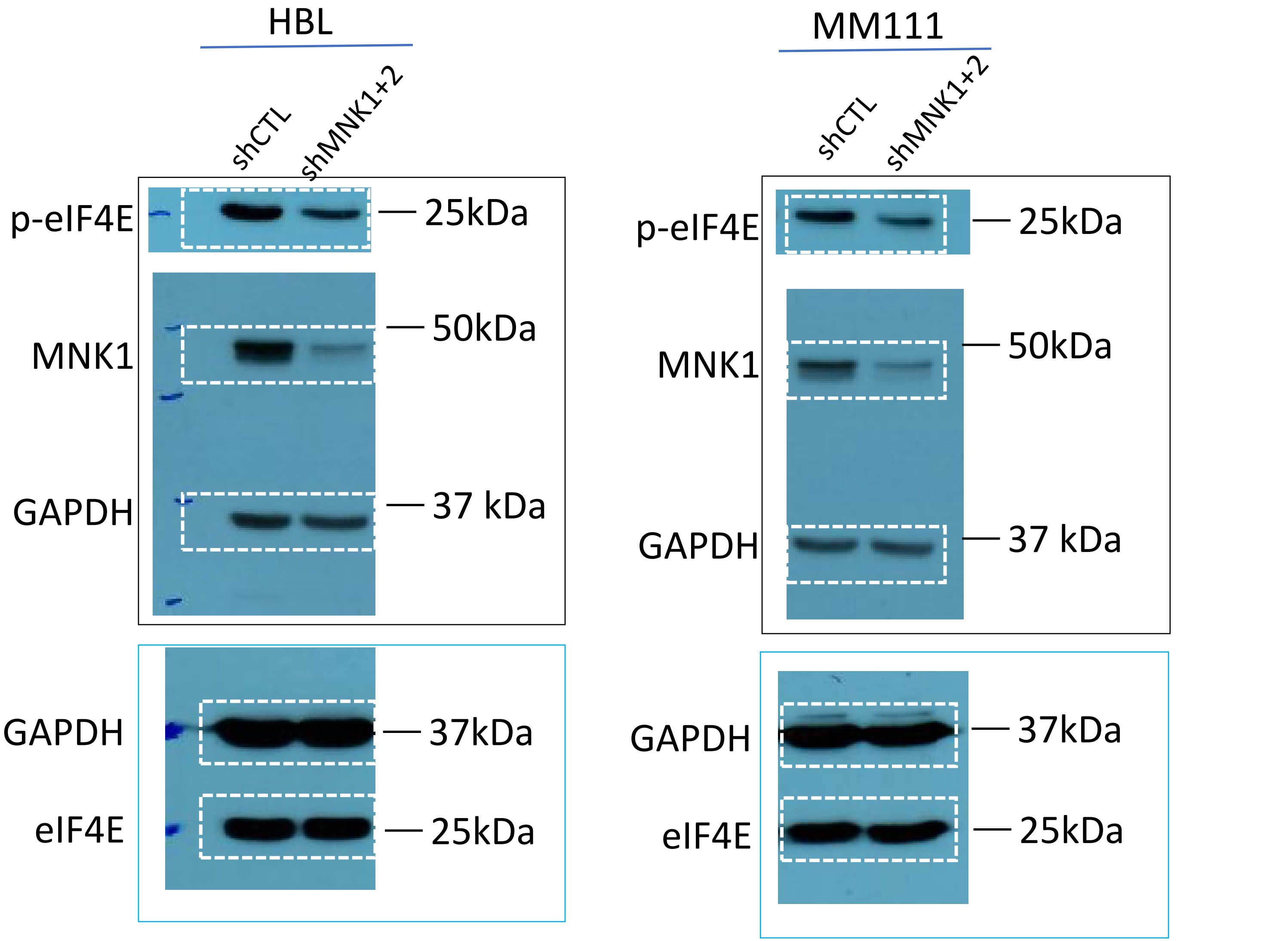
HBL
shCTL
shMNK1+2
p-eIF4E
25kDa
MNK1
50kDa
GAPDH
37 kDa
GAPDH
37kDa
eIF4E
25kDa
MM111
shCTL
shMNK1+2
p-eIF4E
25kDa
MNK1
50kDa
GAPDH
37 kDa
GAPDH
37kDa
eIF4E
25kDa

Figure 2C

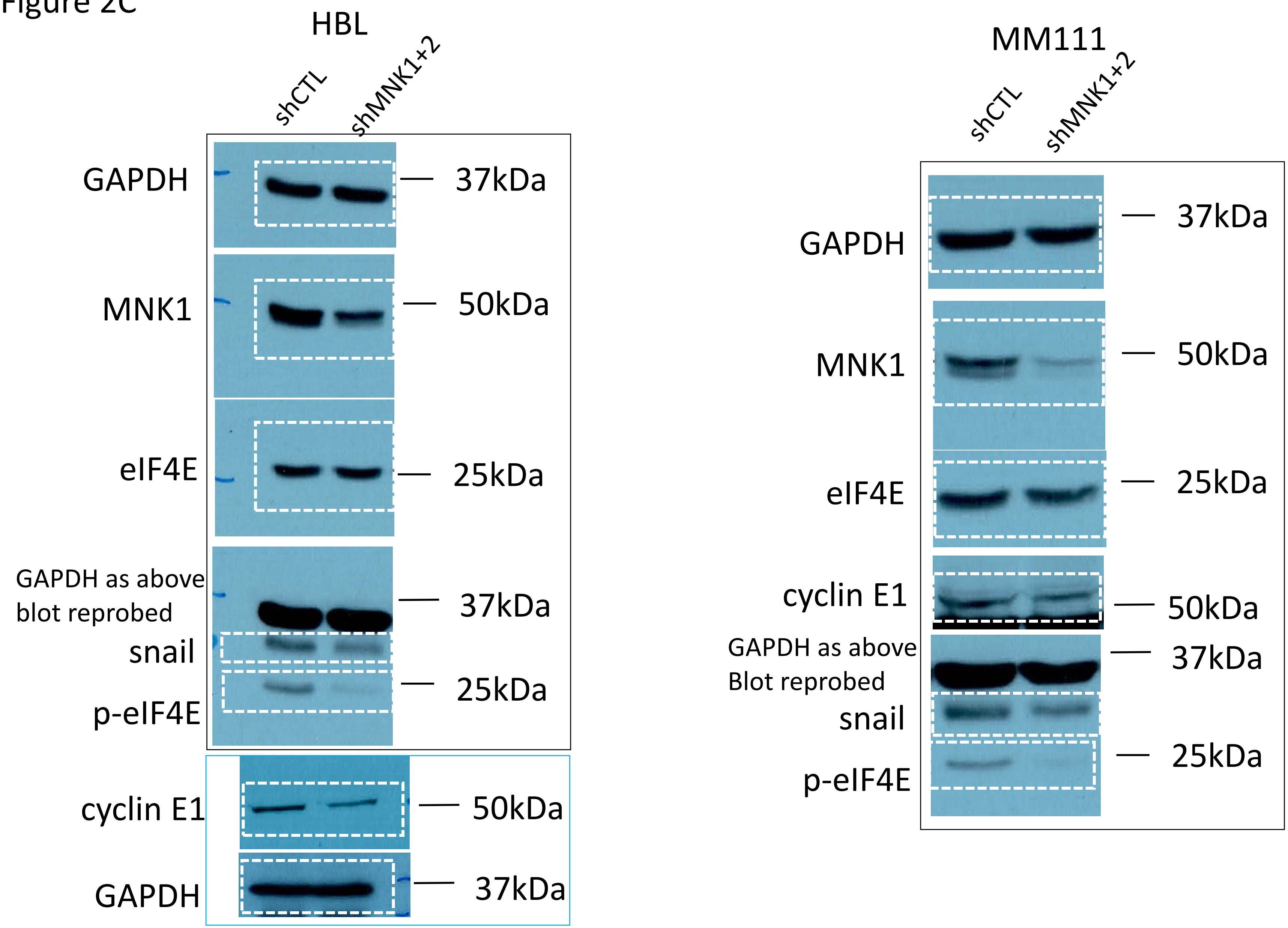

HBL
shCTL
shMNK1+2
GAPDH
37kDa
MNK1
50kDa
eIF4E
25kDa
GAPDH as above
blot reprobed
snail
p-eIF4E
37kDa
25kDa
cyclin E1
50kDa
GAPDH
37kDa
MM111
shCTL
shMNK1+2
GAPDH
37kDa
MNK1
50kDa
eIF4E
25kDa
cyclin E1
50kDa
GAPDH as above
Blot reprobed
snail
p-eIF4E
37kDa
25kDa

Figure 6A

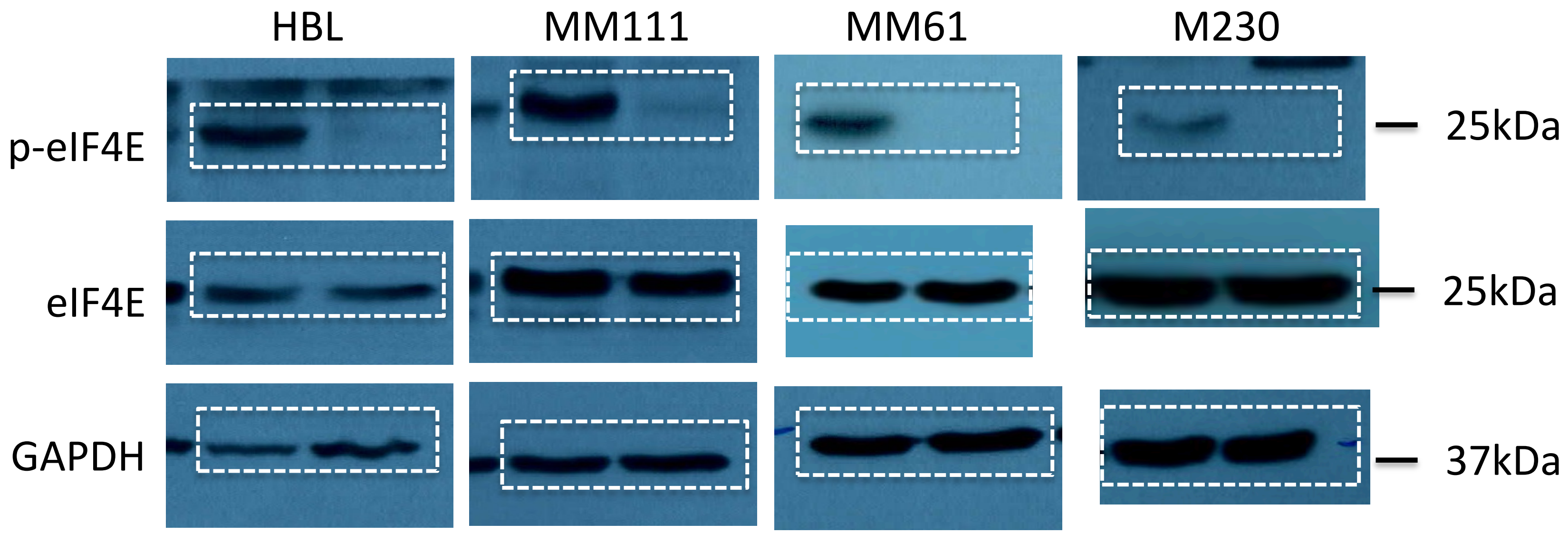

Figure 6B

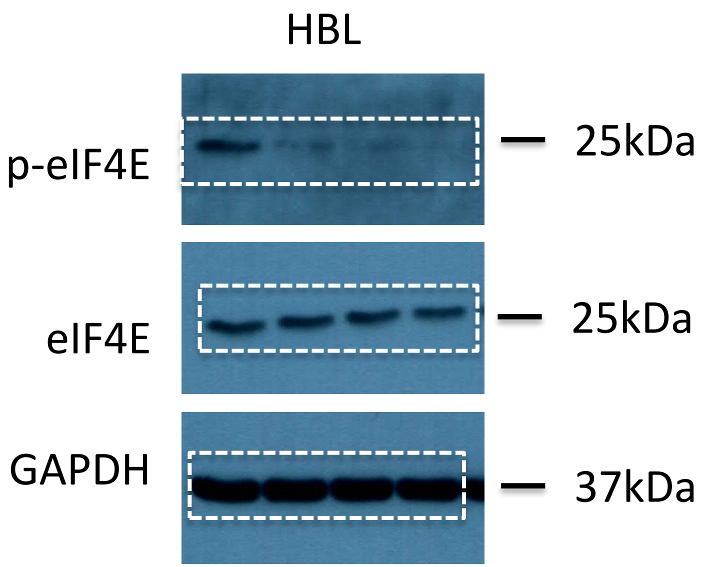

Figure 6C

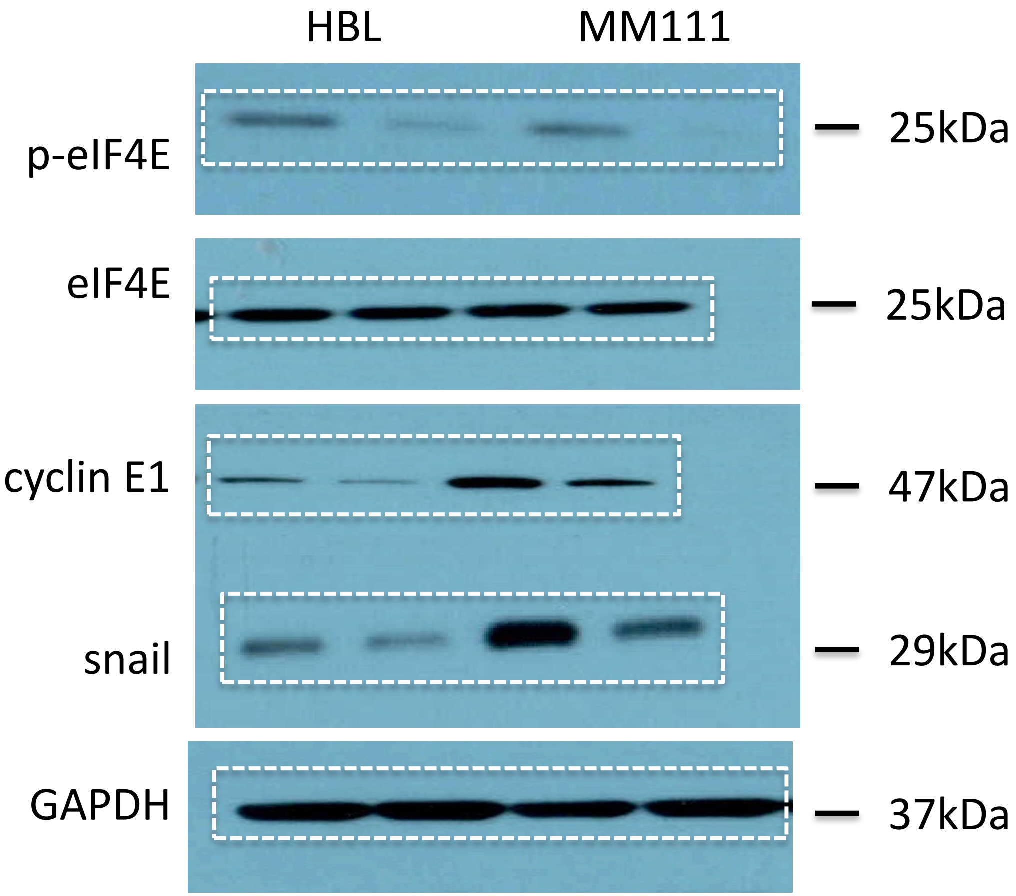
HBL
MM111
p-eIF4E
eIF4E
cyclin E1
snail
GAPDH
25kDa
25kDa
47kDa
29kDa
37kDa

Supplementary Figure S1B

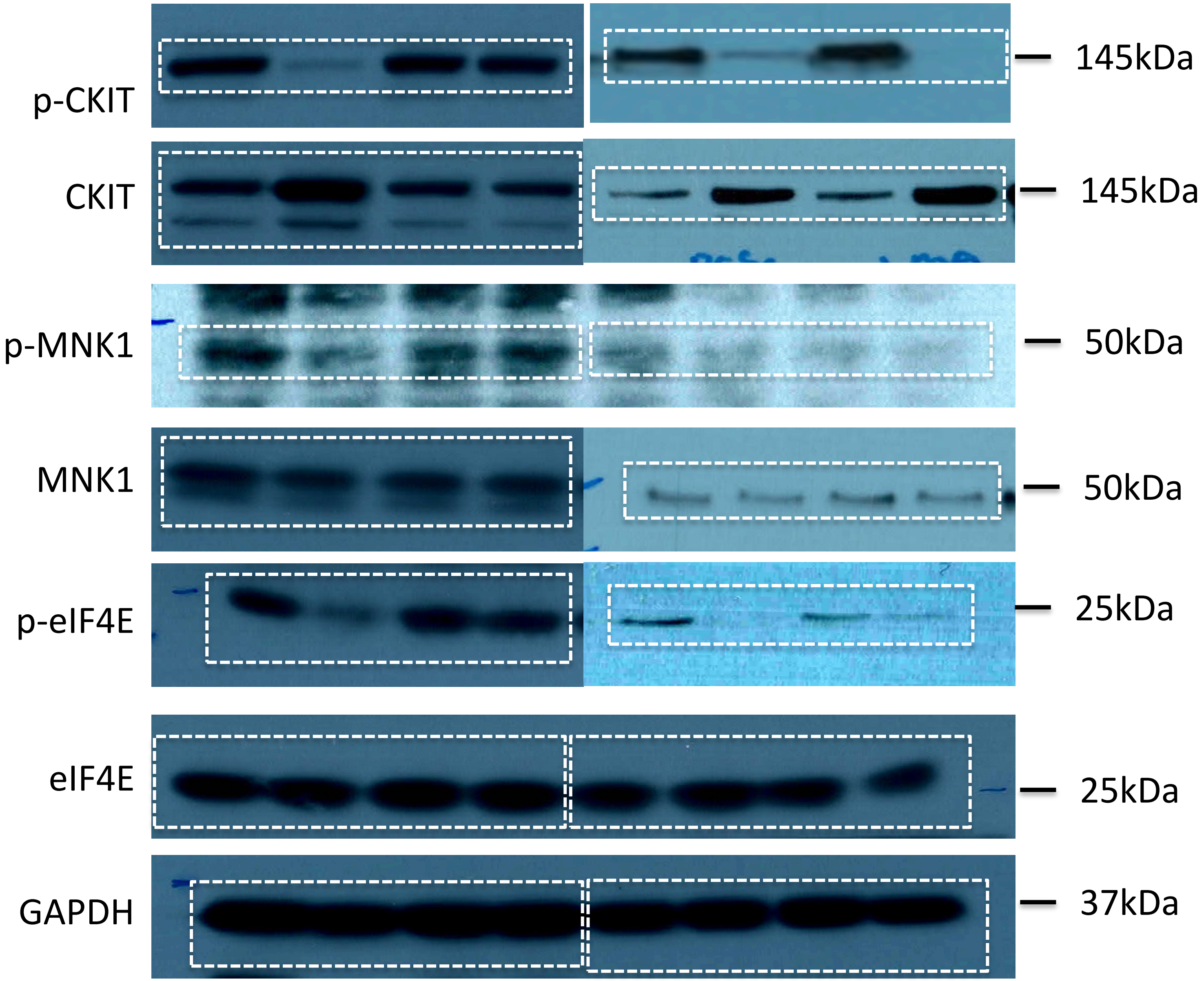

Supplementary Figure S1C

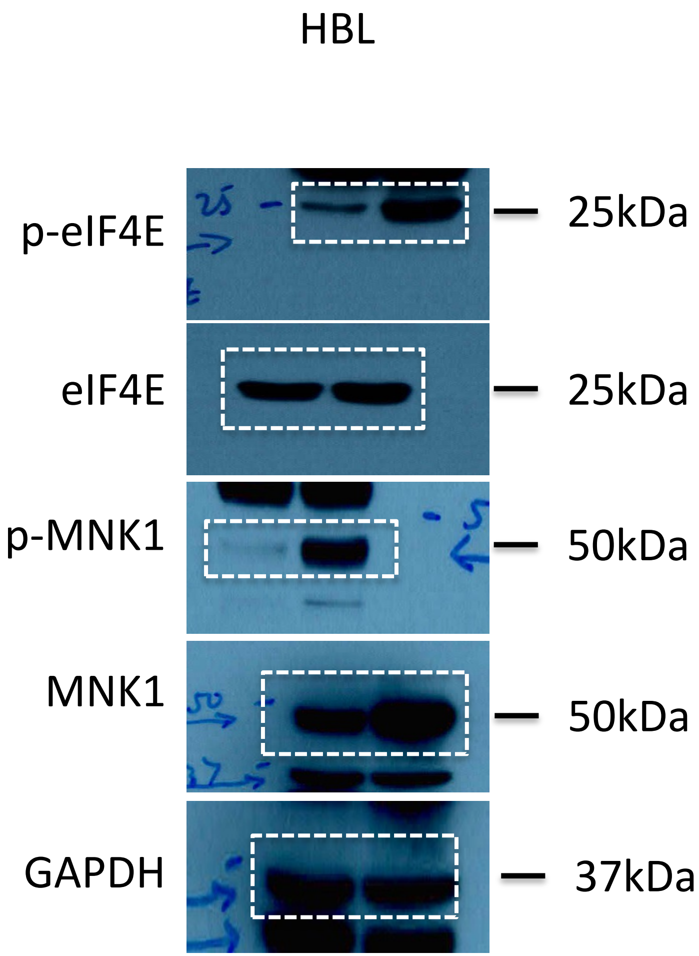

Supplement: Unedited blot and gel images [file jci-134-181338-s239.pdf]
